# High value-added products derived from crude glycerol via microbial fermentation using *Yarrowia* clade yeast

**DOI:** 10.1186/s12934-021-01686-0

**Published:** 2021-10-09

**Authors:** Magdalena Rakicka-Pustułka, Joanna Miedzianka, Dominika Jama, Sylwia Kawalec, Kamila Liman, Tomasz Janek, Grzegorz Skaradziński, Waldemar Rymowicz, Zbigniew Lazar

**Affiliations:** 1 Department of Biotechnology and Food Microbiology, Wroclaw University of Environmental and Life Sciences, Chełmońskiego 37, 51-630 Wroclaw, Poland; 2 Department of Food Storage and Technology, Wroclaw University of Environmental and Life Sciences, Chełmońskiego 37, 51-630 Wroclaw, Poland

**Keywords:** *Yarrowia* clade, Crude glycerol, Polyols, Single cell protein, Single cell oil

## Abstract

**Background:**

Contemporary biotechnology focuses on many problems related to the functioning of developed societies. Many of these problems are related to health, especially with the rapidly rising numbers of people suffering from civilization diseases, such as obesity or diabetes. One factor contributing to the development of these diseases is the high consumption of sucrose. A very promising substitute for this sugar has emerged: the polyhydroxy alcohols, characterized by low caloric value and sufficient sweetness to replace table sugar in food production.

**Results:**

In the current study, yeast belonging to the *Yarrowia* clade were tested for erythritol, mannitol and arabitol production using crude glycerol from the biodiesel and soap industries as carbon sources. Out of the 13 tested species, *Yarrowia divulgata* and *Candida oslonensis* turned out to be particularly efficient polyol producers. Both species produced large amounts of these compounds from both soap-derived glycerol (59.8–62.7 g dm^−3^) and biodiesel-derived glycerol (76.8–79.5 g dm^−3^). However, it is equally important that the protein and lipid content of the biomass (around 30% protein and 12% lipid) obtained after the processes is high enough to use this yeast in the production of animal feed.

**Conclusions:**

The use of waste glycerol for the production of polyols as well as utilization of the biomass obtained after the process for the production of feed are part of the development of modern waste-free technologies.

**Supplementary Information:**

The online version contains supplementary material available at 10.1186/s12934-021-01686-0.

## Background

The *Yarrowia* clade is a group of yeasts consisting of *Candida alimentaria*, *Candida bentonensis*, *Candida hispaniensis*, *Candida pseudorhagii*, *Yarrowia brassicae*, *Yarrowia bubula*, *Yarrowia deformans*, *Yarrowia divulgata*, *Candida galli*, *Candida hollandica*, *Yarrowia lipolytica*, *Candida oslonensis*, *Yarrowia paraphonii*, *Yarrowia phangngaensis*, *Yarrowia porcina*, *Yarrowia yakushimensis*, and *Yarrowia keelungensis*, isolated from different biotic and abiotic environments, e.g. food, sea water, termite intestine [26, 36]. This group of microorganisms is very diverse, and still little is known about their detailed physiology and even less about their full biotechnological potential. Enormous physiological and metabolic diversity exists among the clade members, not only with respect to the different carbon sources used by these species, different growth rates and nutritional requirements, but also differences in protein families (lipases) as well as in protein structure, especially the very important hexokinase responsible for sugar metabolism [12, 34, 45, 48]. Nonetheless, from the biotechnological point of view, the most important difference is in the enormous amount of the metabolite produced by these yeasts [45, 48]. Until now, assimilation of different carbon sources (sugars, free fatty acids, triacylglycerols) and production of lipids by *Yarrowia* clade members have been investigated [34, 36]. The potential of lipid production was further evaluated using cheap substrates such as nondetoxified dilute acid-pretreated switchgrass hydrolysate under highly aerobic conditions [45]. Furthermore, an extensive analysis of production of polyols (mannitol, arabitol, erythritol) and citric acid by the different *Yarrowia* clade species growing in medium containing pure glycerol, fructose and glucose was performed [48]. Very recently, Morin et al. [37] reported the first genetic modification of *Candida hispaniensis* related to the introduction of a heterologous invertase allowing growth on sucrose using a biolistic transformation approach and a dedicated vector.

Single cell protein (SCP), biomass derived from yeast, bacteria, fungi or algae, can be used as a source of protein, mineral elements and vitamins in animal and human nutrition [58]. The proteins from yeast, being the most accepted by consumers, is rich in lysine, leucine, threonine, and valine, as well as including small amounts of cysteine and methionine [19]. The important advantage of SCP production using yeast is not only the utilization of waste or byproducts from other industries but also simultaneous production of high value-added compounds [8]. Apart from potential biosynthesis of SCP, also single cell oil (SCO) can be produced. The SCO, intracellular storage lipids, occurs mostly in the form of triacylglycerols and steryl esters. Yeast can accumulate 20% to 80% of lipids inside the cell under nutrient-limiting conditions (nitrogen or phosphorus) with simultaneous excess of carbon source [9, 28]. Certainly, the development of SCO production technology is also dictated by the controversial discussion over “food or fuel” associated with the utilization of plant oils as substrates for biodiesel production [39]. No less an important aspect related to the use of yeast from the *Yarrowia* clade is their ability to synthesize polyhydroxyalcohols. In recent years, extensive studies have been dedicated to developing biotechnological production of these valuable compounds by *Y. lipolytica* [4, 11, 33, 49, 53]. Replacing sugars with polyols is not an easy task, especially due to the economic aspects. Therefore, searching for new microorganisms and improvement of existing polyol biosynthesis methods lowering the production costs are of high interest.

**Fig. 1:**
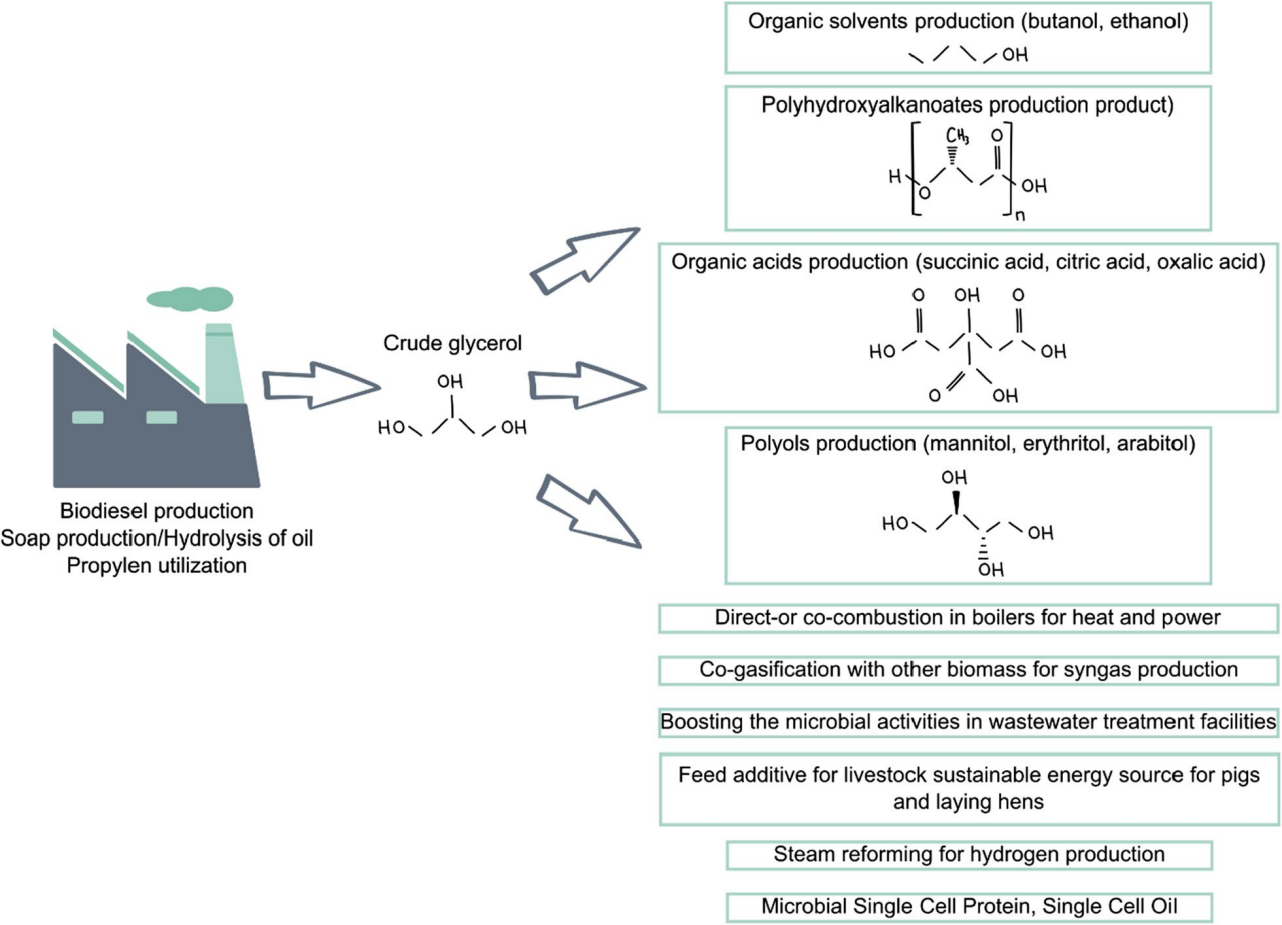
Potential applications of crude glycerol

Every biotechnological process, especially contributing to the circular economy, requires application of cheap and renewable carbon source. One biodegradable industrial feedstock, nowadays frequently applied as a substrate for microorganisms, is crude glycerol generated during biodiesel or soap production (Fig. 1). During 1 ton of biodiesel production up to 100 kg of crude glycerol is generated [16]. To be able to use it in the food, chemicals and cosmetics industries [53], it is necessary to apply expensive purification processes. Crude glycerol instead, due to its large amount of impurities, has limited direct applications (Fig. 1). These limitations were overcome by utilization of raw glycerol as a substrate for microbial fermentation to produce organic solvents, polyhydroxyalkanoates, organic acids as well as polyols (Fig. 1; [53]). Currently, the rapid annual growth of biodiesel production generates large amounts of crude glycerol, also rapidly increasing. For this reason, it has become very important to find an alternative method of its utilization, as the current supply of pure glycerol is sufficient for the industries using it [16]. Development of crude glycerol based processes will also greatly contribute to the undisturbed development of the biodiesel industry [58]. Hence, identification of new microorganisms efficiently using raw glycerol and synthesizing large amounts of value-added chemicals is crucial for developing profitable processes well suited to the circular economy.

In the current study, we investigated the abilities of *Yarrowia* clade yeasts to use raw glycerol derived from the soap and biodiesel industries for biosynthesis of mannitol, arabitol and erythritol. Furthermore, lipid and protein content as well as amino acid composition of the yeast biomass was also evaluated, which shows the great potential of using waste biomass as an ingredient for the production of animal feed. The presented technology shows great potential of *Yarrowia* clade members to recycle waste compounds, reduce their negative environmental impact, and to reduce the overall costs associated with waste management.

## Results and discussion

As mentioned in the introduction, the capacity of the *Yarrowia* clade yeasts for polyhydroxyalcohol production has already been analyzed [48]. In that study, however, pure substrates were used as carbon sources (glucose, fructose and glycerol). As expected, similarly to *Y. lipolytica*, other clade members produced polyols mainly using glycerol, whereas sugars were not a good substrate for biosynthesis of sweeteners. It is mainly related to the osmotic pressure caused by glycerol. Although polyol biosynthesis by *Yarrowia* clade members was analyzed using pure glycerol, to fulfill the expectations of the circular economy and develop sustainable processes, in the current study crude glycerol, a waste product from two industries, the production of biodiesel and the production of soap, was investigated.

### Growth of *Yarrowia* clade on crude glycerol

**Fig. 2:**
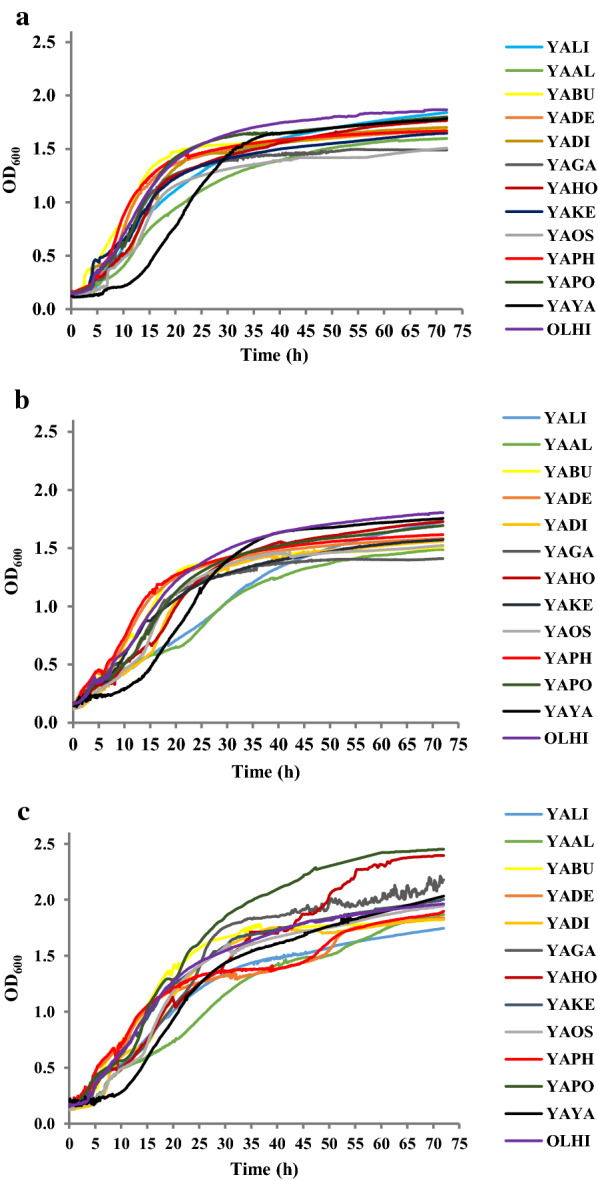
Growth curves of *Yarrowia* clade yeast growing on: **a** pure glycerol, **b** crude glycerol from biodiesel industry or **c** crude glycerol from soap production. Triple experiments were performed at 28 °C under constant agitation using microplate reader

First, the ability of different *Yarrowia* clade species to grow in a medium with different sources of waste glycerol was investigated. The characteristics of the different carbon sources are presented in Table 1. In the reference experiment, all the strains were cultured in medium with pure glycerol (Fig. 2a). In this control experiment, no growth inhibitors, such as salts or heavy metals, were present in the medium. Furthermore, the most studied yeast, *Y. lipolytica*, known to grow well on glycerol, was used as a reference species here and throughout the entire study. Some differences in the growth profiles of the different species could be observed. The slowest performing species or species with a long lag phase were found to be YAYA and YAAL, but the final OD_600_ of these yeasts was similar to other clade members (Fig. 2a).

**Table 1:** Characteristics of glycerol used in this study

Name	GLY 1	GLY 2	GLY 3
Company	Poch, Gliwice, Poland	ORLEN Południe, Trzebinia, Poland	Grupa Azoty, Chorzów, Poland
Origin	Waste from biodiesel production	Waste from biodiesel production	Waste from soap production
Purity (%)	98	87	80
Water % (m/m)	0.5	15	3.6
Methanol % (m/m)	0	0.3	0.2
MONG (matter organic non glycerol) % (m/m)	0	6	4.0
NaCl % (m/m)	0	0	7.59
Methanol % (m/m)	0	0.3	0.2
Nitrogen content	0	0.023	0.041
Ash	0	0.93	8.76
Cu (mg/kg)	0	0.096	0.66
Mg (mg/kg)	0	1.68	5.45
Fe (mg/kg)	0	1.99	26.42
Zn (mg/kg)	0	0.09	1.30
K (mg/kg)	0	4388.16	231.32
Na (mg/kg)	0	422.85	31,632.89
Cl (mg/kg)	0	1200	46,000
Ca (mg/kg)	0	40.27	52.36

Similarly to the medium with pure glycerol, also in YNB medium based on crude glycerol from the biodiesel industry, YAYA and YAAL showed slow or delayed growth (Fig. 2b). Surprisingly, also A101 grew significantly slower than other clade members, which was not as clearly observed in pure glycerol (Fig. 2b). The largest differences, mostly in growth curves of the *Yarrowia* clade members, were observed when crude glycerol from the soap industry was used as a carbon source (Fig. 2c). This substrate was highly polluted by salt and MONG (matter organic nonglycerol) (Table 1). However, the contaminations present in this crude glycerol did not weaken the growth of yeast cells. Unlike previous cultures, two strains exhibited very different growth profiles than other species, YAPO and YAHO (Fig. 2c), and reached the highest final OD_600_ among the tested clade members. Once again, YAYA and YAAL exhibited long or delayed growth. Furthermore, this substrate caused a diauxic growth profile for some of the species (YADE, YAHO, YAPH, also the slowly growing YAAL). Moreover, OLHI was one of the species which grew very well in medium with every analyzed glycerol.

### Polyol synthesis by *Yarrowia* clade yeast during the shake-flask experiment

**Fig. 3:**
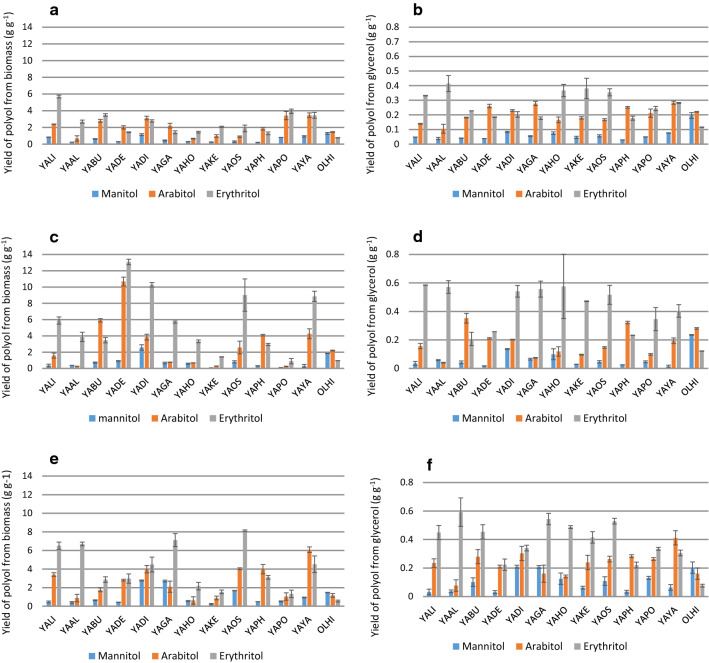
Yield of polyol production from: **a**, **b** biomass growing on pure glycerol, **c**, **d** crude glycerol from biodiesel industry and **e**, **f** crude glycerol from soap production by yeast belonging to *Yarrowia* clade during shake-flask experiment

In the next step, biosynthesis of polyols was analyzed in shake flask cultures using crude glycerol. Pure substrate of technical grade was used in a control experiment. Very high diversity could be observed among the *Yarrowia* clade members depending on the substrate used (Fig. 3). Interestingly, the yields of erythritol and arabitol (both from the biomass and from the substrate) were higher than the same parameters obtained for mannitol (Fig. 3a, b). Until now, the best known species belonging to the *Yarrowia* clade, *Y. lipolytica*, is known to produce mostly erythritol and mannitol [47, 60]. The highest erythritol yield from biomass was noted for YALI, YABU, YAPO, YAYA (Fig. 3a), whereas the highest erythritol yield from pure glycerol was observed for different species, YAAL, YAKE, YAHO and YAOS and ranged from 0.35 to 0.41 g g^−1^ (Fig. 3b). In turn, the best arabitol producers were YAYA, YAPO and YADI strains, for which the yield from biomass was higher than 3.0 g g^−1^ (Fig. 3a). The highest arabitol yield from pure glycerol was observed for YAYA (0.28 g g^−1^). Mannitol production was significant only for the OLHI strain, where the yield from glycerol reached 0.2 g g^−1^ (Fig. 3b). It should be pointed out here that in the analyzed conditions, for YADE, YADI, YAGA, YAPH and OLHI the product yield from the consumed substrate for arabitol was the highest among the three polyols. This phenomenon was observed for the first time and suggests different kinetic characteristics of the enzymes from the pentose phosphate pathway among clade members.

Very interesting results for the analyzed species were obtained when pure glycerol was substituted by the crude one. Using raw glycerol from the biodiesel industry, the polyol ratio changed (Fig. 3c, d). YADI, YAGA, YAPO and YAYA were characterized by a clear predominance of erythritol biosynthesis (Fig. 3d) compared to the medium with pure glycerol (Fig. 3b). Furthermore, seven strains produced more than 0.4 g g^−1^ of erythritol (Fig. 3d), which was the highest value when pure glycerol was used as a substrate (Fig. 3b). It was interesting that YABU significantly increased the biosynthesis of arabitol, while OLHI was again the only species with a significant amount of mannitol synthesized (Fig. 3d). Moreover, the amount of polyols produced from the biomass reached much higher values when crude glycerol from the biodiesel industry was used (Fig. 3c). Yield much higher than the one obtained for YALI (*Y. lipolytica*, a control species) were observed for YABU, YADI, YAOS, YAYA, while YABU also produced the highest amount of arabitol at the same time (Fig. 3c). The obtained data confirmed that polyol biosynthesis strongly depends on the composition of the medium, which in our study was strongly connected to the components in raw glycerol.

Lower polyol biosynthesis was observed when crude glycerol from soap production was used as a carbon source (Fig. 3e, f). Only three species produced more than 0.5 g g^−1^ of erythritol—YAAL, YAGA, YAOS (Fig. 3f). Two species significantly changed their profile of polyols secreted. Of these, raw glycerol YABU produced much more erythritol than arabitol (ARA:ERY = 0.62 for glycerol from the soap industry, whereas ARA:ERY = 1.67 was observed from glycerol obtained from the biodiesel industry). The opposite situation was noted for YAYA, which secreted more arabitol than erythritol (ARA:ERY = 1.32 for soap derived glycerol and ARA:ERY = 0.48 for biodiesel derived glycerol). Furthermore, three species (YADI, YAGA, OLHI) secreted mannitol in a significant amount as its yield exceeded 0.2 g g^−1^. Taking into account the yield of erythritol from biomass, four species deserve attention—YALI, YAAL, YAGA, YAOS—whereas considering arabitol yield from biomass YAYA stands out clearly (Fig. 3e). The different production of polyols in the medium with crude glycerol from the soap industry compared to the biodiesel-derived glycerol is very likely caused by the presence of sodium chloride in the former. For *Y. lipolytica*, the optimal concentration of sodium chloride to induce appropriate osmotic pressure for erythritol biosynthesis is 2.5% [60]. However, in the current study, a similar concentration of total polyols was observed for YALI when both crude glycerols were applied as carbon sources (Y_P/S_ = 0.78 g g^−1^ for biodiesel derived glycerol and 0.72 g g^−1^ for soap derived glycerol; Fig. 3d, f). In contrast, lower amounts of these compounds were obtained from pure glycerol (Y_P/S_ = 0.52 g g^−1^, Fig. 3b). This difference is probably due to the presence of additional components present in crude glycerol (see “Methods”). It is worth pointing out that no organic acids were produced during the shake flask cultures.

Summarizing the observations, the species belonging to *Yarrowia* clade proved to have promising potential for efficient polyol biosynthesis using crude glycerol as a carbon source. Comparing the present results to data available in the literature, erythritol yield obtained for YAHO and YAAL (0.57 g g^−1^) is quite high and was not observed previously for any known species during flask cultures. The well-characterized species *Y. lipolytica*, when cultivated in flask cultures, secreted 20–35 g dm^−3^ of erythritol with a yield ranging from 0.33 to 0.42 g g^−1^ [60]. The best yield obtained in a flask scale experiment reported 0.52 g g^−1^ of erythritol for *Y. lipolytica* strain ACA-YC 5030 [42]. A similar type of culture for *Candida magnoliae* allowed only 0.08 g g^−1^ of this valuable compound to be obtained [21]. A very valuable result obtained in this study was the large amount of arabitol produced by clade members, especially YABU, YAPH and YAYA. It is the first report of *Yarrowia* yeast secreting this polyol in this amount; however, to further improve it, optimization of media components and culture parameters is needed. So far, strains of *Debaryomyces hansenii* have been among the best arabitol producers, especially SBP-1 (NRRL Y-7483), reaching 0.5 g g^−1^ with initial glycerol concentration of 150 g dm^−3^ [23].

### Polyols as value-added chemicals synthesized from crude glycerol by *Yarrowia* clade on bioreactor scale

**Fig. 4:**
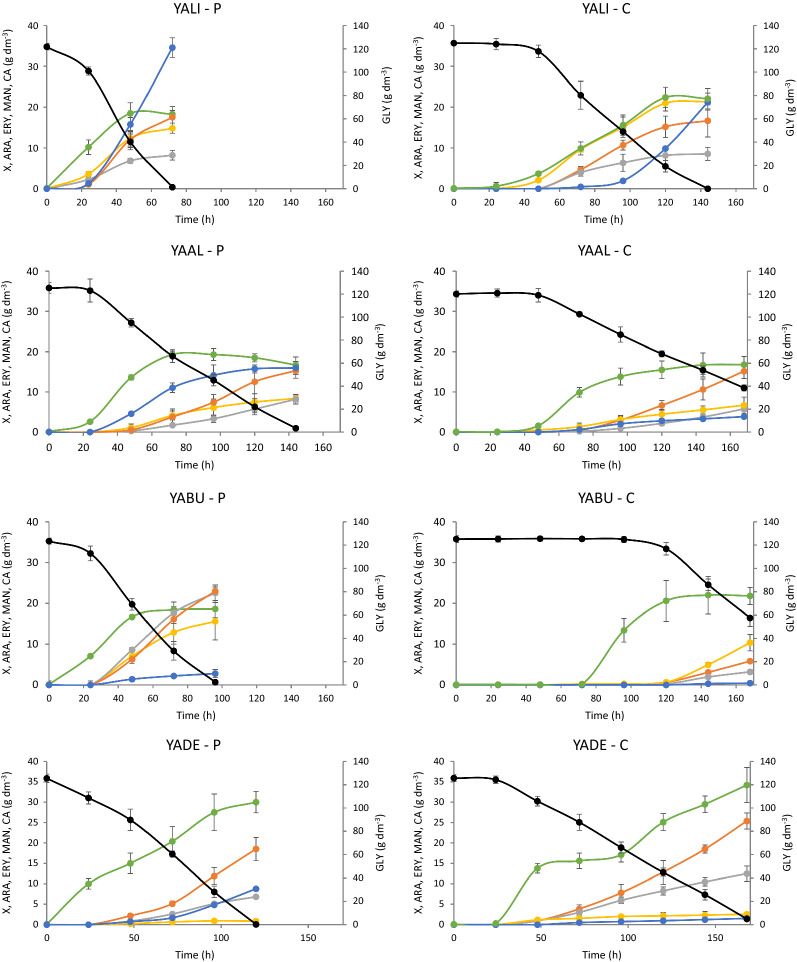

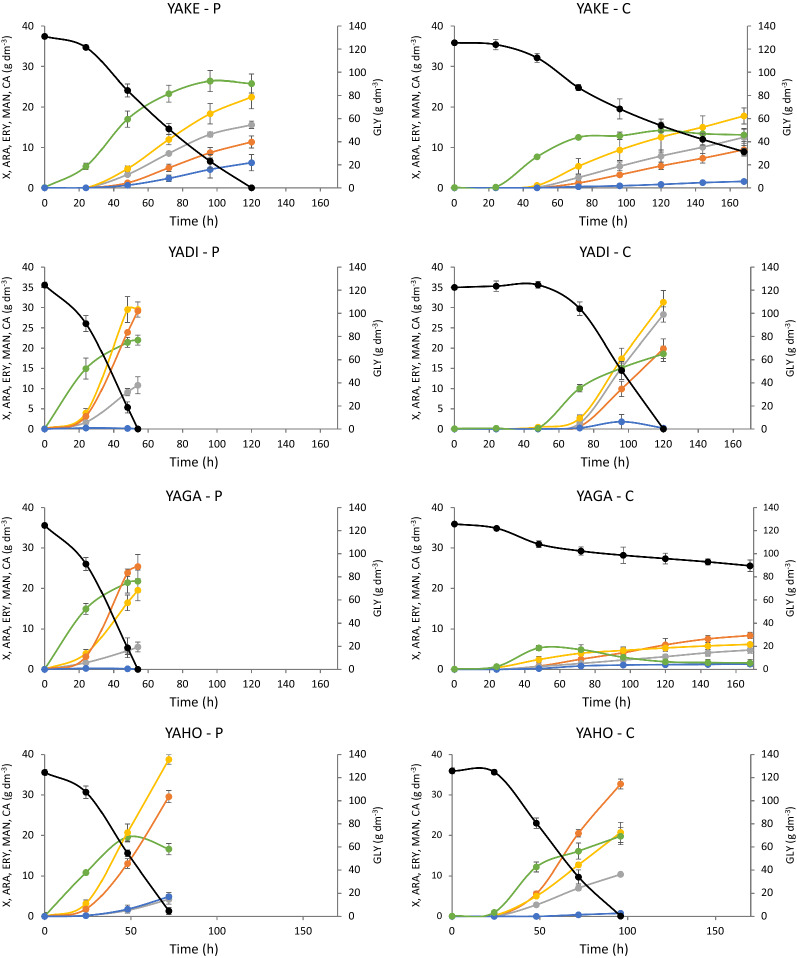

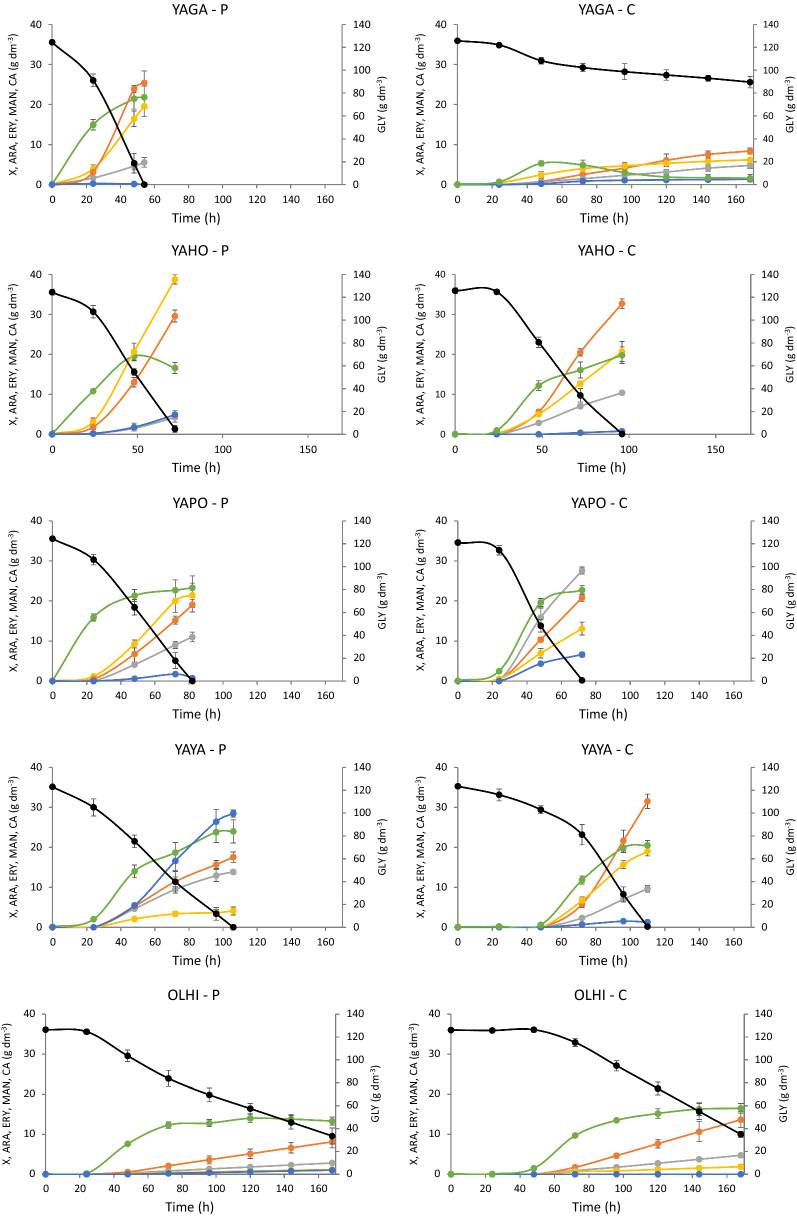
Kinetics of growth (●), glycerol consumption (●) as well as arabitol (●), erythritol (●), mannitol (●) and citric acid (●) secretion by *Yarrowia* clade members during bioreactor cultures with pure glycerol (P) or crude glycerol from biodiesel industry (C). All experiments were performed in triplicate

In the next step of the research, all strains belonging to the *Yarrowia* clade were analyzed for polyol secretion on a larger scale. Cultures were performed in 5 dm^3^ bioreactors in a working volume of 2 dm^3^. Two glycerol types were used—technical as well as glycerol from biodiesel production. Figure 4 represents the main parameters characterizing the performed cultures of all analyzed species growing in medium with either pure or crude glycerol. Not only were differences among all analyzed species observed but also very large differences were noted when the same species was growing in pure or crude glycerol. The cultures on pure glycerol lasted from 54 h for YAGA and YADI to 168 h for OLHI strains (Fig. 4). The duration of cultures using crude substrate in many cases was extended. The only species requiring 72 h to consume all available glycerol when growing in crude glycerol based medium was YAPO. The highest concentration of biomass (approximately 30 g dm^−3^) was achieved by YAPH and YADE growing in pure glycerol, whereas YADE reached 34.2 g dm^−3^ when growing in crude glycerol (Fig. 4). YAGA turned out to be the species growing the worst when crude glycerol from the biodiesel industry was used as a carbon source (Fig. 4). This species also showed the worst growth in our previous studies in medium containing NaCl [48]. It is sensitive to NaCl and/or other mineral salts which might be present in the crude glycerol.

**Fig. 5:**
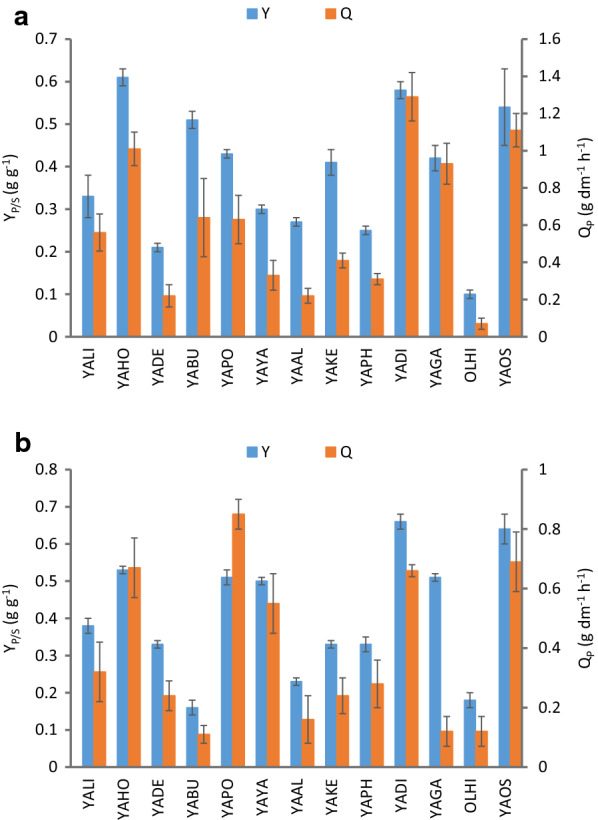
Yield and productivity of total polyol (erythritol, arabitol and mannitol) biosynthesis by the yeast belonging to *Yarrowia* clade growing on pure (**a**) and crude glycerol (**b**) from biodiesel industry in bioreactor cultures

All parameters characterizing bioreactor cultures with pure and crude glycerol from the biodiesel industry are summarized in Fig. 5 and Additional file 1: Table S1. The best producers of polyols from crude glycerol, where the sum of mannitol, arabitol and erythritol was the highest, were YADI (79.46 g dm^−3^) and YAOS (76.78 g dm^−3^). Interestingly, the same species growing in medium with pure substrate secreted 10 or more g dm^−3^ less polyols (Additional file 1: Table S1). Among the other species, YAHO also deserves attention due to the high concentration of polyols produced. This species secreted 72.62 g dm^−3^ growing on pure glycerol and 63.84 g dm^−3^ from the crude substrate. These strains produced large amounts of polyols on both substrates, whereas the remaining species were characterized by high production of polyols on one substrate only. YADI, YAOS and YAHO also showed the highest yield of polyol biosynthesis, which exceeded 0.6 g g^−1^ (Fig. 5). Among all analyzed species, OLHI was found to be the weakest polyol producer. It secreted not more than 20 g dm^−3^ of polyols regardless of the used glycerol.

Each of the analyzed yeast species was characterized by a different profile of polyols produced. Differences in the amount of each individual polyol produced were also observed depending on the substrate used for the same species (Fig. 5, Additional file 1: Table S1). The best erythritol yield was noted for YAHO (0.33 g g^−1^) when crude glycerol was used as a carbon source. However, the highest productivity of the same polyol was reached by YADI (0.54 g dm^−3^ h^−1^) growing on the same substrate. The best arabitol producer from crude glycerol was YAOS (0.25 g g^−1^), whereas the same polyol was secreted much better by YABU (0.2 g g^−1^) when pure glycerol was used. In general, most of the analyzed species secreted less arabitol when pure glycerol was applied. In turn, mannitol secretion was the highest for YAHO, YAYA, YAGA and YAOS and ranged from 0.22 to 0.26 g g^−1^ (Fig. 5, Additional file 1: Table S1), whereas it should be noted that YAGA grew poorly and its final mannitol concentration reached only 8.4 g dm^−3^. For all the studied species, the only byproduct produced during the fermentation was citric acid. Interestingly, the concentration of citric acid was higher when pure glycerol was used as a carbon source and a significant amount of it was secreted only for YALI, YAAL and YAYA (Fig. 4). Other species either produced less than 10 g dm^−3^ of this compound or no citric acid secretion was observed.

Based on the available literature data, the best known and the most efficient polyol-producing species is *Y. lipolytica* [47]. A genetically engineered strain of *Y. lipolytica* overexpressing the *SUC2* gene from *S. cerevisiae* (invertase) together with the native *GUT1* gene (glycerol kinase) produced polyols with the yield of 0.67 g g^−1^ from sucrose and glycerol [47]. Similar results were obtained in the current study for YADI growing on crude glycerol from the biodiesel industry (0.66 g g^−1^). Furthermore, most of the available information about polyol production, especially erythritol biosynthesis, comes from studies on *Y. lipolytica* or *C. magnoliae* [32]. The high erythritol yield, described in the literature, obtained for different *Y. lipolytica* strains ranged from 0.5 to 0.6 g g^−1^ in batch or fed-batch cultures [4, 15, 50, 61]. Another erythritol producer, *C. magnoliae*, showed lower erythritol yield noted even for fed-batch cultures (~ 0.4 g g^−1^) [24, 51]. The same species is also able to produce mannitol. In shake flask culture *C. magnoliae* secreted 67 g dm^−3^ of mannitol from fructose (~ 0.45 g g^−1^; [56]). Although microbiological production of mannitol is not as popular as erythritol, it is known that apart from *C. magnoliae* also *Candida zeylanoides*, *Torulopsis mannitofaciens* and *Torulopsis versalitis* are able to produce this compound [13, 40, 56]. Even less popular is the microbiological production of arabitol. Good arabitol producers are the yeasts *Debaryomyces hansenii* and *Candida parapsilosis*, which secretes 0.51–0.54 g g^−1^ of this compound [25, 30]. The best arabitol producers in the current study, YADI and YAOS, yielded 0.24–0.25 g g^−1^ of this polyol. All polyols produced by selected *Yarrowia* clade species were produced in quite high yields, especially from the crude glycerol from the biodiesel industry. This is a very good starting point for optimization of medium composition and culture parameters as well as for genetic engineering of the metabolic pathways in this species. So far, there are no available genetic tools for that yeast; however, developing efficient modification methods will most certainly allow further enhanced biosynthesis of these compounds by *Yarrowia* clade species.

### *Yarrowia* clade yeast as a potential SCP and SCO producing microorganism

**Fig. 6:**
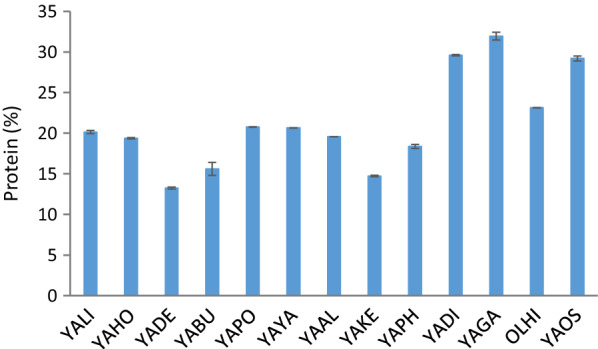
Protein content of the yeast biomass of *Yarrowia* clade species growing on crude glycerol from biodiesel industry in bioreactor cultures

**Fig. 7:**
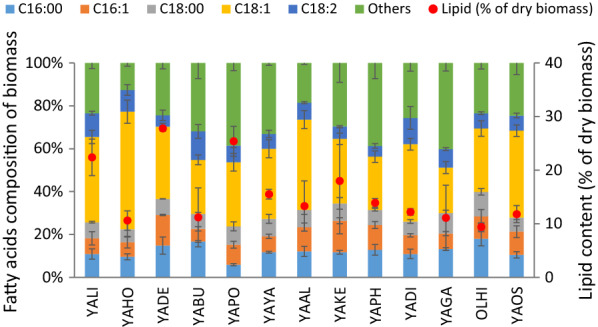
Lipid content (% of dry biomass) and fatty acid composition of the yeast biomass of *Yarrowia* clade species growing on crude glycerol in bioreactor cultures

The composition of *Yarrowia* clade yeast, obtained after cultivation on crude glycerol from the biodiesel industry, as protein and lipid content as well as amino acid composition, was analyzed. All results are summarized in Figs. 6 and 7 and in Additional file 2: Table S2. The amount of protein in the yeast biomass of clade members has never been studied so far; however, it represents very valuable knowledge, especially in terms of considering these species as safe for industrial use (GRAS status). The biomass of *Yarrowia* clade species differed remarkably in the protein content—from 13.2 to 31.9% for YADE and YAGA, respectively (Fig. 6). Similarly to polyol production, protein content can be compared to studies producing fodder yeast using *Y. lipolytica*. Juszczyk et al. [19] growing the *Y. lipolytica* S11 strain in medium with pure glycerol obtained very high protein content (50.1%). In the current study, the protein content was much lower and reached only 9.4% for the YALI strain A101 (Fig. 6). The same strain cultured in medium supplemented with lipids and selenium produced biomass with 56.4% protein [38]. Nonetheless, despite the large amount of nitrogen in the polyol production medium, it was not an optimal medium for biomass production. Due to that, the benefit here is the use of the produced biomass as a byproduct from polyol production and its utilization for fodder yeast manufacturing. To increase the protein content in the biomass of *Yarrowia* clade species more studies should be performed focused on sufficient supply of inorganic and organic sources of nitrogen.

The biomass was also further analyzed for its amino acid composition (Additional file 2: Table S2). In the reference protein (the amino acid composition of protein of whole egg), isoleucine, leucine, lysine, methionine/cysteine, phenylalanine/tyrosine, threonine, tryptophan and valine are present [17, 62]. The composition of proteins produced by *Yarrowia* clade species is represented by all the essential amino acids (except tryptophan, which was not analyzed in our study) in a reasonable concentration (Additional file 2: Table S2). Moreover, the composition of biomass of *Yarrowia* clade species was rich in aspartic and glutamic acids, alanine and lysine and poor in serine, methionine and cysteine. The composition of *Y. lipolytica* proteins obtained in the current study was comparable to the previously reported amino acid composition of this species in different industrial processes [18, 35, 47]. Furthermore, similar composition of proteins was noted for S*accharomyces cerevisiae* or *Candida utilis*, which have already been applied for the production of feed [1].

To further explore the nutritional value of *Yarrowia* clade biomass, lipid content and fatty acid composition were determined (Fig. 7). The highest lipid content was noted in the biomass of YADE, YAPO and YALI and reached more than 22%. It used to be the case that yeasts accumulating lipids in more than 20% of their biomass were considered as oleaginous [3]. It has already been verified that some of the *Yarrowia* clade members are able to accumulate a large amount of lipids inside the cells in both pathways, ex novo—the best species being YAHI [36]—and de novo—the best being YAPH [45]. Apart from the substrate used, one of the key parameters for lipid biosynthesis is the carbon to nitrogen ratio [6, 7, 42]. In general, efficient SCO production requires on one hand to maximize cell growth and, on the other hand nitrogen limitation, while avoiding its starvation [44]. Lipid production by *Y. lipolytica* strains is well documented in the literature [5, 6, 31, 41, 46] and the most abundant fatty acids occurring in its lipids are: oleic acid (C18:1), palmitic acid (C16:0), stearic acid (C18:0), and palmitoleic acid (C16:1). In our study, three species showed more than 20% lipid presence in the biomass—YALI, YADE and YAPO (Fig. 7). In all cases, the dominant fatty acid was C18:1; however, YABU, YAPO, YAPH and YAGA produced very large amounts of other fatty acids in their pool of lipids. It is important to mention here that a large amount of lipids produced by cells in a process of polyol production is another benefit for using the residual biomass for feed manufacturing, especially using non-engineered strains.

### Cluster analysis of polyol, protein and lipid production within *Yarrowia* clade members

**Fig. 8:**
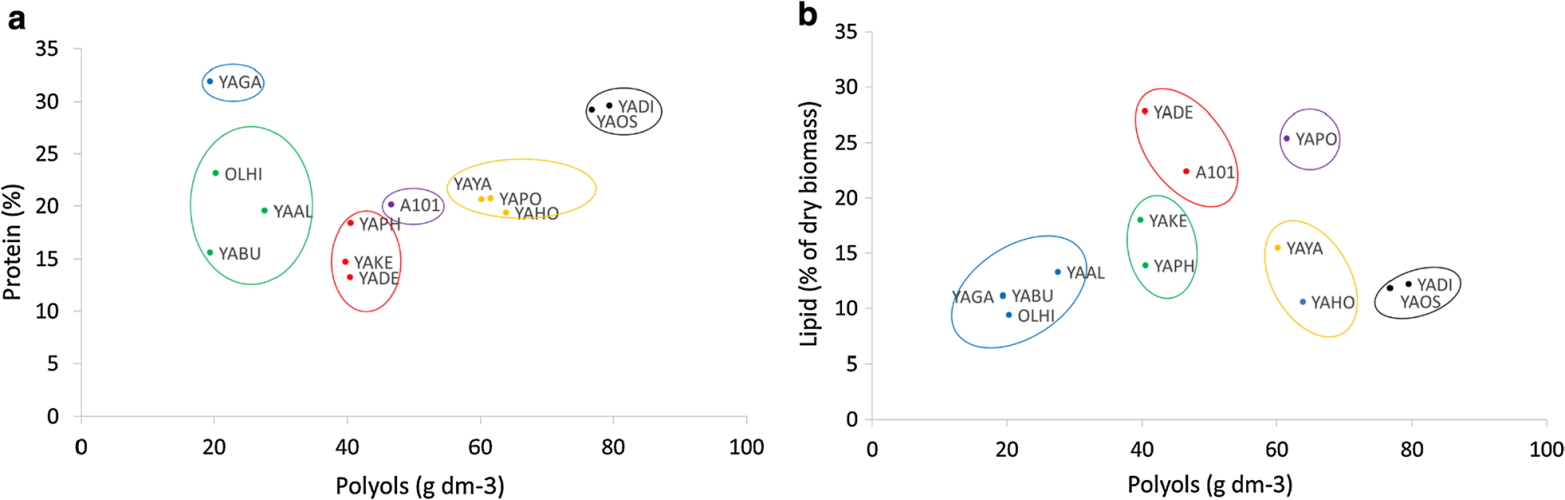
Cluster analysis of: **a** protein content and polyol concentration, as well as **b** lipid content and polyol concentrations for *Yarrowia* clade yeast growing on crude glycerol-based medium during bioreactor cultures

Results obtained during polyol production using crude glycerol from biodiesel production as a carbon source were subject to cluster analysis (Fig. 8). “Polyols”, “Protein” and “Lipid” were taken as variables and Ward’s hierarchical agglomerative clustering method, grouping statistical data into so-called clusters having minimal internal diversity, was applied. Figure 8 presents cluster analysis in two systems of variables, “Polyols” vs. “Protein” (Fig. 8a) and “Polyols” vs. “Lipid” (Fig. 8b). Six clusters were obtained. Taking into account polyols and proteins, YADI and YAOS turned out to be very attractive species for further research (Fig. 8a). An attractive species, when polyols and lipids were taken into account, turned out to be YAPO (Fig. 8b). In both cases, YAAL, YABU and OLHI were the weakest species (Fig. 8).

### Value-added chemicals produced using crude glycerol from soap production

**Fig. 9:**
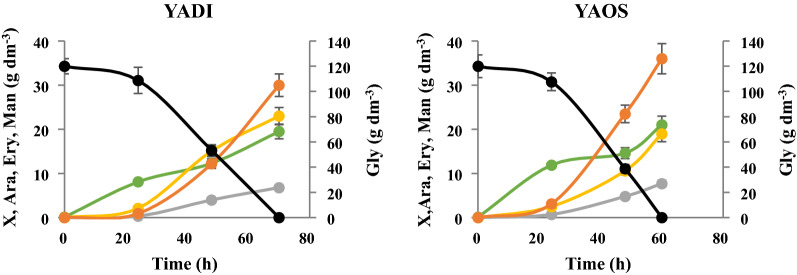
Kinetics of growth (●), glycerol consumption (●) as well as arabitol (●), erythritol (●) and mannitol (●) secretion by *Y. divulgata* (YADI) and *C. oslonensis* (YAOS) during bioreactor cultures with crude glycerol from soap industry. All experiments were performed in triplicate

Based on the results from the clustering analysis, YADI and YAOS were chosen for analysis of polyol production using crude glycerol from soap production. Both strains were cultured in bioreactors under the same conditions as for the cultures with crude glycerol from the biodiesel industry. The culture of YAOS was 10 h shorter than the one of YADI; however, both species produced similar amount of biomass—around 20 g dm^−3^ (Fig. 9). The same species cultured under the same conditions on crude glycerol from biodiesel production produced a slightly smaller amount of biomass (Fig. 4). However, higher biomass production resulted in a lower concentration of polyols produced (Table 2) compared to culture biodiesel derived glycerol (Fig. 5). Both species produced around 60 g dm^−3^ of polyols with a yield reaching 0.50–0.52 g g^−1^ with mannitol being predominant. Both species showed similar protein and lipid content. Crude glycerol from soap production changed these parameters. The protein content was lower and the concentration of lipids was higher, compared to the biomass obtained from crude glycerol from biodiesel production (Table 2, Fig. 6). The crucial enzymes (glycerol kinase, glycerol-3-phosphate dehydrogenase, transketolase and erythrose reductase) from the erythritol biosynthesis pathway were examined. All investigated enzymes were active during the whole culture. Glycerol kinase showed the highest activity after 24 h of culture for YAOS and it decreased at the stationary phase, except for YADI, where the activity of glycerol kinase remained almost at the same level (Additional file 3: Table S3). The erythrose reductase is so far considered as a determining step in the production of erythritol. In the current study the highest activity of erythrose reductase (0.323 U mg^−1^) during the growth phase was reported for the YADI strain (Additional file 3: Table S3). The differences in polyols production between the same strains growing on different glycerols, especially higher erythritol concentration using raw glycerol, may be caused by changes in the concentration of Fe^2+^ and Cu^2+^ (Table 1). During erythritol biosynthesis by *C. magnoliae*, the addition of Zn^2+^, Fe^2+^, and Ca^2+^ resulted in a slight increase of erythritol concentration, while supplementation with Cu^2+^ and Mn^2+^ inhibited erythritol formation [54]. In the work of Tomaszewska et al. [61], no inhibitory effect of the divalent copper, iron, manganese, and zinc ions on the production of erythritol from glycerol by *Y. lipolytica* was observed. Savergave et al. [54], reported, that copper ions caused a reduction of arabitol and mannitol secretion, whereas manganese, iron, and zinc ions resulted in increased amounts of these components in comparison to erythritol.

**Table 2:** The yield and productivity of polyols biosynthesis by *Y. divulgata* and *C. oslonensis* on crude glycerol from soap production in bioreactor cultures

Species	SUM_P_	Y_P/S_	Y_ERY/S_	Y_ARA/S_	Y_MAN/S_	Q_P_	Q_ERY_	Q_ARA_	Q_MAN_	Lipid	Protein
	(g dm^−3^)	(g g^−1^)				(g dm^−3^ h^−1^)				(% of dry biomass)	(%)
YADI	59.8 ± 2.8	0.50 ± 0.03	0.19 ± 0.01	0.06 ± 0.01	0.25 ± 0.02	0.85 ± 0.05	0.33 ± 0.01	0.10 ± 0.01	0.43 ± 0.02	19.6 ± 0.9	19.65 ± 0.10
YAOS	62.7 ± 4.0	0.52 ± 0.04	0.16 ± 0.02	0.06 ± 0.01	0.30 ± 0.01	1.05 ± 0.04	0.32 ± 0.02	0.13 ± 0.01	0.60 ± 0.03	18.6 ± 1.3	24.22 ± 0.40

SUM_P_: sum of polyols (arabitol, mannitol, erythritol); Y_P/S_: yield of polyols from glycerol; Y_ERY/S_: yield of erythritol production from glycerol; Y_ARA/S_: yield of arabitol production from glycerol; Y_MAN/S_: yield of mannitol production from glycerol; Q_P_: productivity of polyols; Q_ERY_: productivity of erythritol; Q_ARA_: productivity of arabitol; Q_MAN_: productivity of mannitol

## Conclusions

Contemporary biotechnology focuses on many problems related to the functioning of developed societies. Many of the aforementioned problems are related to health, especially with the spiraling numbers of people suffering from civilization diseases, such as obesity or diabetes. An increased supply of sucrose has a particularly noticeable negative effect on health. In recent years, polyols have gained particular attention as table sugar substitutes. They are particularly desirable due to their low calorific value and the possibility of their production using microorganisms from renewable waste substrates.

In the current study, the already known potential of the yeast belonging to the *Yarrowia* clade for polyol production was further proved using crude glycerol obtained from the biodiesel and soap industries. *Yarrowia divulgata* and *Candida oslonensis* turned out to be particularly interesting species, as they produced the largest amount of polyols using all tested glycerol types. Furthermore, both species also produced a significant amount of biomass, which is an interesting by-product from the polyol production process. The obtained biomass was characterized by quite large amounts of proteins and lipids, which can be an attractive substrate for the production of fodder yeast. The amino acid composition of the proteins contained in the biomass contains all the necessary exogenous amino acids. Both the use of waste glycerol for the production of polyols and utilization of the biomass obtained after the process for the production of feed are part of the development of modern waste-free technologies. These results lay the foundation for future advances in polyol biosynthesis using different renewable raw materials by the means of genetic engineering and process optimization.

## Methods

### Strains

In this study the following strains belonging to the *Yarrowia* clade were used: OLHI—*Candida hispaniensis* CBS9996, YAAL—*Candida alimentaria* CBS10151, YABU—*Yarrowia bubula* CBS12934, YADE—*Yarrowia deformans* CBS2071, YADI—*Yarrowia divulgata* CBS11013, YAGA—*Candida galli* CBS9722, YAHO—*Candida hollandica* CBS4855, YALI—*Yarrowia lipolytica A-101*, YAOS—*Candida oslonensis* CBS10146, YAPH—*Yarrowia phangngaensis*, CBS10407, YAPO—*Yarrowia porcina* CBS12932, YAYA—*Yarrowia yakushimensis* CBS10253 and YAKE—*Yarrowia keelungensis* CBS11062. The strains were commercially purchased from Westerdijk Fungal Biodiversity Institute, Royal Netherlands Academy of Arts and Sciences (KNAW), Netherlands and were isolated from various biotic and abiotic environments. The species form the *Yarrowia* clade and were identified using a vast number of techniques such as sequence analyses of the D1/D2 domain and the ITS region, PCR-mediated fingerprint analyses, DNA–DNA reassociation and mating experiments [2, 10, 22, 26, 27, 29, 43]. All strains were kept at − 80 °C in 25% (v/v) glycerol solution. One day before use, cells were inoculated on YPD plates and incubated overnight at 28 °C.

### Substrate

The characteristics of substrates used in the study are listed in Table 1.

### Medium and culture conditions

YPD medium containing 20 g dm^−3^ Bactopeptone (Merck, Germany), 10 g dm^−3^ yeast extract (Merck, Germany), 20 g dm^−3^ glucose (Merck, Germany) was used for growing the biomass before use.

### Microcultures

The growth of *Yarrowia* clade species was tested in the Microplate Reader, BioTek Instruments, Inc. (Winooski, Vermont, USA) in YNB medium with 5 g dm^−3^ of NH_4_Cl (Yeast Nitrogen Base, Sigma) and 2% (w/v) of glycerol (GLY1, GLY2, GLY3). The precultures were grown for 24 h in YPD medium at 28 °C, 180 rpm, followed by centrifugation. Cells were washed twice with sterile distilled water and the optical density (OD_600_) of the inoculum was standardized to 10. Growth profiles were analyzed in a 96-well plate containing 190 µL of the medium and 10 µL of the inoculum. Cells were grown for 72 h at 22 °C with continuous shaking and analysis of OD_600_ every 15 min. The experiments were performed in triplicate for each strain.

### Shake flask experiment

The differences in polyol biosynthesis using three types of glycerol were tested using the shake flasks and the medium consisted of: carbon source (GLY1, GLY2, GLY3)—100 g; (NH_4_)_2_SO_4_—2.7 g; KH_2_PO_4_—0.2 g; MgSO_4_ 7H_2_O—1 g; yeast extract—1.6 g; in 1 dm^3^ of tap water [48]. The precultures were grown in YP medium containing the same carbon source (2%) as the main culture (GLY1, GLY2, GLY3) for 48 h in a 250 cm^3^ flask on a rotary shaker at 28 °C and 140 rpm. Precultures were harvested at OD_600_ between 5 and 7. Flasks containing 50 cm^3^ of the main polyol production medium were inoculated to an initial OD_600_ of 0.25 and cultivated at 28 °C in 250 cm^3^ Erlenmeyer flasks at 200 rpm for 6 days. The experiment was performed in three biological replicates for each strain.

### Bioreactor studies

Mannitol, arabitol and erythritol production was also analyzed in bioreactor cultures in medium containing: carbon source (GLY1, GLY2)—100 g; (NH_4_)_2_SO_4_—2.7 g; KH_2_PO_4_—0.2 g; MgSO_4_·7H_2_O—1 g; yeast extract—1.6 g; in 1 dm^3^ of tap water. The precultures were grown in 100 cm^3^ YP medium with 2% of the corresponding glycerol for 72 h in 300 cm^3^ flasks on a rotary shaker at 28 °C and 140 rpm. Precultures were harvested, the biomass was washed twice with sterile distilled water and the initial optical density (OD_600_) in the bioreactor was adjusted to 0.5. All bioreactor cultures were performed in a 5 dm^3^ stirred-tank reactor (BIOSTATB-PLUS, Sartorius, Germany) with the working volume of 2.0 dm^3^ at 28 °C (22 °C for YAAL and YAKE). Aeration and stirring rates were set at 0.8 vvm and 800 min^−1^, respectively. pH 3 was maintained automatically by additions of 20% (w/v) NaOH solution. All cultures were run until complete exhaustion of the carbon source. A bioreactor with the appropriate medium was sterilized in an autoclave at 121 °C for 20 min. All cultures were conducted in three biological replicates and standard deviations were calculated.

### Analytical methods

During shake flask experiment, 10 cm^3^ of cultures was collected at the end of the process. Samples (10 cm^3^) from bioreactor cultures were taken every 24 h. The samples were centrifuged (5 min, 5000 rpm). The biomass was washed with distilled water and filtered on 0.45 µm pore size membranes. The biomass was determined gravimetrically after drying at 105 °C and expressed in g dm^−3^. The concentrations of glycerol, erythritol, mannitol, arabitol, citric acid and α-ketoglutaric acid were measured in the supernatants using high performance liquid chromatography (Dionex-Thermo Fisher Scientific, UK) with a HyperRez Carbohydrate H+ column (Thermo Scientific, Waltham, MA) coupled to a UV (k = 210 nm) and a refractive index (RI) detector (Shodex, Ogimachi, Japan). The column was eluted with 25 mM trifluoroacetic acetic acid with a rate of 0.6 cm^3^ min^−1^ at 65 °C.

The residual biomass after the bioreactor processes was subjected to lipid, protein and amino acids composition analysis. The amino acid composition of samples was determined by ion-exchange chromatography after 23 h of hydrolysis with 6 N HCl at 110 °C. After cooling, filtering and washing, the hydrolyzed sample was evaporated in a vacuum evaporator at a temperature below 50 °C. The dry residue was dissolved in a buffer of pH 2.2. The prepared sample was analyzed using the ninhydrin method [55, 57]. The pH 2.6, 3.0, 4.25, and 7.9 buffers were applied. The ninhydrin solution was buffered at pH 5.5. The hydrolyzed amino acids were determined using an AAA-400 analyzer (INGOS, Prague, Czech Republic). A photometric detector was used, working at two wavelengths, 440 nm and 570 nm. A column of 350 × 3.7 mm, packed with ion exchanger Ostion LG ANB (INGOS), was used. Column temperature was kept at 60–74 °C and detector temperature at 121 °C. The calculations were carried out relative to an external standard. No analysis of tryptophan was carried out.

Lipids determination was conducted by the two-step direct transesterification method. 1 mg of biomass was collected, centrifuged for 2 min at 8000 rpm and the supernatant was discarded. Next, 100 µL of C17:0 internal standard (Sigma-Aldrich, St. Louis, MO, USA) was added to the samples. After addition of 500 µL of 0.5 M sodium methoxide, the samples were vortexed for 60 min at room temperature. Following incubation, 40 µL of anhydrous H_2_SO_4_ and 1 mL of hexane were added and left overnight at room temperature. After centrifugation at 8000 rpm for 1 min the upper hexane layer containing fatty acid methyl esters (FAMEs) was collected and analyzed by a GC–MS instrument (Shimadzu, Kyoto, Japan) equipped with a Zebron ZB-FAME capillary column (30 m × 0.25 mm × 0.20 µm). The samples (1 µL at 250 °C) were injected in splitless mode using helium (1 mL min^−1^). Identification of fatty acids was carried out by comparison of retention times with reference compounds (Supelco 37 Component FAME Mix, Sigma-Aldrich).

### Enzymatic assays

The key enzyme activities were measured in the growth phase (after 24 h of culture) and at the end of the production phase (96 h) for YAOS and YADI strains, according to Tomaszewska et al. [63]. Activities of the enzymes important for erythritol biosynthesis [glycerol kinase—GK, (EC 2.7.1.30) [20], glycerol-3-phosphate dehydrogenase—GPDH, (EC 1.1.1.8) [64], transketolase—TK, (EC 2.2.1.1) [59] and erythrose reductase—ER, (EC 1.1.1.21)] were determined in the supernatants [14]. One unit (U) of the enzyme activity was defined as 1 µmol of NADH/NADPH consumed or produced within 1 min (λ = 340 nm) at the reaction conditions. The enzyme activities were expressed as U per mg of protein (U mg^−1^ of protein) and as U per 100 mg samples of cell dry weight (U CDW^−1^). The assay for each strain was carried out in three biological replicates (bioreactor culture) and three technical replicates and standard deviations were calculated.

### Calculation of fermentation parameters

The yield of sum of polyols, arabitol, erythritol and mannitol from glycerol (Y_P,_ Y_ERY_, Y_ARA_, Y_MAN_), expressed in g g^−1^, was calculated using Eq. (1) [52]:

1 $$Y_{P} = \frac{P}{S}.$$

The yield of polyols, arabitol, erythritol, mannitol production from biomass (Y_X_), expressed in g g^−1^, was calculated using Eq. (2) [52]:

2 $$Y_{X} = \frac{P}{X}.$$

The productivity of total polyols, arabitol, erythritol and mannitol (Q_P_, Q_ARA,_ Q_ERY_, Q_MAN_), expressed in g dm^−3^ h^−1^, was calculated using Eq. (3) [52]:

3 $$Q = \frac{P}{t}.$$

In all these equations, P denotes total polyols, arabitol, erythritol, mannitol concentration in the culture broth at the end of the cultivations (g dm^−3^), S indicates the total amount of glycerol consumed (g dm^−3^), t denotes duration of the fermentation process (h), and X represents biomass concentration (g dm^−3^).

Cluster analysis (Ward’s method) was used to find homologous groups among the strains when polyol and protein concentrations and polyol and lipid contents in bioreactor cultures on glycerol from the biodiesel industry were taken into account. Squared Euclidean distance was taken as a measure of proximity between strains. Results of the study were analyzed statistically with GNU Octave 4.3.0+ software using two functions. The first of them returns the Euclidean distance between any two rows in the input data. The second function produces a hierarchical clustering dendrogram using the Ward method that defines the way the distance between two clusters is computed. In this case, it is the sum of squared deviations about the group mean.

## Supplementary Information

**Additional file 1: Table S1.** The yield and productivity of erythritol, arabitol and mannitol biosynthesis by the yeast belonging to *Yarrowia* clade growing on pure and crude glycerol from biodiesel industry in bioreactor cultures.

**Additional file 2: Table S2.** Amino acids composition of the yeast biomass of *Yarrowia* clade species growing on crude glycerol from biodiesel industry in bioreactor cultures.

**Additional file 3: Table S3.** Comparison of enzymatic activities of key enzymes from erythritol biosynthesis pathway from glycerol by selected stains from the *Yarrowia* clade.

## Data Availability

The datasets used and/or analyzed during the current study are available from the corresponding author on reasonable request.

## Abbreviations

OLHI: *Candida hispaniensis* CBS9996
YAAL: *Candida alimentaria* CBS10151
YABU: *Yarrowia bubula* CBS12934
YADE: *Yarrowia deformans* CBS2071
YADI: *Yarrowia divulgata* CBS11013
YAGA: *Candida galli* CBS9722
YAHO: *Candida hollandica* CBS4855
YALI: *Yarrowia lipolytica A-101*
YAOS: *Candida oslonensis* CBS10146
YAPH: *Yarrowia phangngaensis*, CBS10407
YAPO: *Yarrowia porcina* CBS12932
YAYA: *Yarrowia yakushimensis* CBS10253
YAKE: *Yarrowia keelungensis* CBS11062
