# Supplementary material for: High value-added products derived from crude glycerol via microbial fermentation using Yarrowia clade yeast
Source: Microb Cell Fact. 2021 Oct 9;20:195. doi: 10.1186/s12934-021-01686-0 (PMC8502345; doi:10.1186/s12934-021-01686-0)
Supplement: Supplementary file 1 — Additional file 1: Table S1. The yield and productivity of erythritol, arabitol and mannitol biosynthesis by the yeast belonging to Yarrowia clade growing on pure and crude glycerol from biodiesel industry in bioreactor cultures. [file 12934_2021_1686_MOESM1_ESM.docx]

**Table S1.** The yield and productivity of erythritol, arabitol and mannitol biosynthesis by the yeast belonging to *Yarrowia* clade growing on pure and crude glycerol from biodiesel industry in bioreactor cultures.

| Strain | YALI | YAHO | YADE | YABU | YAPO | YAYA | YAAL | YAKE | YAPH | YADI | YAGA | OLHI | YAOS |
| --- | --- | --- | --- | --- | --- | --- | --- | --- | --- | --- | --- | --- | --- |
| Pure glycerol | | | | | | | | | | | | | |
| SUM_P_ | 40.62±1.24 | 72.62±6.87 | 26.24±3.42 | 61.17±4.56 | 51.54±5.78 | 35.50±10.23 | 31.83±7.45 | 49.43±11.34 | 29.76±8.97 | 69.51±3.87 | 50.49±8.56 | 12.13±3.67 | 64.23±7.54 |
| Y_ERY/S_ | 0.13±0.01 | 0.34±0.03 | 0.01±0.00 | 0.11±0.04 | 0.18±0.05 | 0.03±0.01 | 0.07±0.01 | 0.19±0.03 | 0.02±0.01 | 0.25±0.07 | 0.16±0.02 | 0.01±0.00 | 0.23±0.06 |
| Y_ARA/S_ | 0.05±0.03 | 0.04±0.01 | 0.06±0.02 | 0.20±0.03 | 0.09±0.01 | 0.12±0.05 | 0.07±0.01 | 0.12±0.04 | 0.06±0.01 | 0.09±0.01 | 0.05±0.01 | 0.03±0.01 | 0.09±0.02 |
| Y_MAN/S_ | 0.15±0.02 | 0.23±0.04 | 0.14±0.04 | 0.20±0.02 | 0.16±0.06 | 0.15±0.03 | 0.13±0.01 | 0.10±0.02 | 0.17±0.03 | 0.24±0.01 | 0.21±0.05 | 0.06±0.01 | 0.22±0.05 |
| Q_ERY_ | 0.21±0.07 | 0.54±0.09 | 0.01±0.00 | 0.16±0.04 | 0.26±0.07 | 0.04±0.01 | 0.06±0.01 | 0.19±0.03 | 0.02±0.00 | 0.55±0.02 | 0.36±0.09 | 0.01±0.00 | 0.48±0.03 |
| Q_ARA_ | 0.10±0.02 | 0.06±0.01 | 0.06±0.03 | 0.23±0.05 | 0.13±0.04 | 0.13±0.01 | 0.06±0.01 | 0.13±0.02 | 0.08±0.02 | 0.2±0.06 | 0.1±0.01 | 0.02±0.01 | 0.18±0.07 |
| Q_MAN_ | 0.24±0.09 | 0.41±0.07 | 0.15±0.03 | 0.24±0.05 | 0.23±0.1 | 0.17±0.09 | 0.11±0.03 | 0.09±0.01 | 0.21±0.04 | 0.54±0.05 | 0.47±0.03 | 0.05±0.01 | 0.45±0.03 |
| Crude glycerol from biodiesel industry | | | | | | | | | | | | | |
| SUM_P_ | 46.59±4.54 | 63.84±5.92 | 40.41±2.67 | 19.42±3.98 | 61.52±2.63 | 60.14±4.24 | 27.60±4.67 | 39.75±6.45 | 40.5±4.76 | 79.46±3.54 | 19.4±5.76 | 20.29±4.35 | 76.78±5.67 |
| Y_ERY/S_ | 0.17±0.02 | 0.17±0.01 | 0.02±0.02 | 0.17±0.02 | 0.11±0.01 | 0.16±0.02 | 0.08±0.01 | 0.20±0.02 | 0.04±0.01 | 0.26±0.01 | 0.16±0.01 | 0.01±0.00 | 0.13±0.02 |
| Y_ARA/S_ | 0.07±0.03 | 0.10±0.01 | 0.11±0.01 | 0.05±0.01 | 0.23±0.01 | 0.08±0.01 | 0.07±0.01 | 0.14±0.01 | 0.12±0.01 | 0.24±0.01 | 0.13±0.01 | 0.01±0.01 | 0.25±0.01 |
| Y_MAN/S_ | 0.13±0.01 | 0.27±0.01 | 0.22±0.02 | 0.09±0.01 | 0.17±0.01 | 0.26±0.01 | 0.19±0.01 | 0.11±0.01 | 0.18±0.01 | 0.17±0.01 | 0.22±0.01 | 0.16±0.01 | 0.26±0.01 |
| Q_ERY_ | 0.15±0.05 | 0.22±0.08 | 0.02±0.04 | 0.06±0.01 | 0.18±0.04 | 0.17±0.04 | 0.04±0.02 | 0.11±0.04 | 0.03±0.01 | 0.26±0.07 | 0.04±0.01 | 0.01±0.00 | 0.14±0.02 |
| Q_ARA_ | 0.06±0.01 | 0.11±0.02 | 0.07±0.02 | 0.02±0.01 | 0.4±0.07 | 0.09±0.01 | 0.03±0.01 | 0.07±0.01 | 0.10±0.06 | 0.24±0.08 | 0.03±0.01 | 0.03±0.01 | 0.27±0.03 |
| Q_MAN_ | 0.12±0.06 | 0.34±0.06 | 0.15±0.04 | 0.03±0.01 | 0.29±0.05 | 0.29±0.06 | 0.09±0.01 | 0.06±0.01 | 0.15±0.04 | 0.17±0.05 | 0.05±0.02 | 0.08±0.02 | 0.28±0.01 |
| Y_ERY/S_ – yield of erythritol production in g g^-1^; Y_ARA/S_ – yield of arabitol production in g g^-1^; Y_MAN/S_ – yield of mannitol production in g g^-1^; Q_P_ – productivity of polyols in g dm^-3^ h^-1^; Q_ERY_ – productivity of erythritol in g dm^-3^ h^-1^; Q_ARA_ – productivity of arabitol g dm^-3^ h^-1^; Q_MAN_ – productivity of mannitol in g dm^-3^ h^-1^ | | | | | | | | | | | | | |
