# Supplementary material for: High value-added products derived from crude glycerol via microbial fermentation using Yarrowia clade yeast
Source: Microb Cell Fact. 2021 Oct 9;20:195. doi: 10.1186/s12934-021-01686-0 (PMC8502345; doi:10.1186/s12934-021-01686-0)
Supplement: Supplementary file 2 — Additional file 2: Table S2. Amino acids composition of the yeast biomass of Yarrowia clade species growing on crude glycerol from biodiesel industry in bioreactor cultures. [file 12934_2021_1686_MOESM2_ESM.docx]

**Table S2.** Amino acids composition of the yeast biomass of *Yarrowia* clade species growing on crude glycerol from biodiesel industry in bioreactor cultures.

| **Strain** | **YALI** | **YAHO** | **YADE** | **YABU** | **YAPO** | **YAYA** | **YAAL** | **YAKE** | **YAPH** | **YADI** | **YAGA** | **OLHI** | **YAOS** |
| --- | --- | --- | --- | --- | --- | --- | --- | --- | --- | --- | --- | --- | --- |
| mg/g protein | | | | | | | | | | | | | |
| aspartic acid | 20.2±0.2 | 20.5±0.4 | 11.3±0.2 | 15.5±0.4 | 20.2±0.2 | 19.4±0.4 | 19.3±0.4 | 14.7±0.3 | 16.8±0.1 | 30.5±0.1 | 30.6±0.4 | 18.4±0.1 | 29.7±0.3 |
| threonine | 20.4±0.4 | 14.8±0.3 | 13.1±0.1 | 17.2±0.1 | 19.3±0.2 | 12.5±0.7 | 15.0±0.1 | 9.9±0.2 | 10.5±0.1 | 20.8±0.2 | 21.0±0.1 | 12.1±0.1 | 19.2±0.1 |
| serine | 14.0±1.4 | 9.9±0.1 | 8.8±0.2 | 8.5±0.4 | 9.6±0.5 | 9.4±0.1 | 10.7±0.1 | 8.5±0.1 | 8.6±0.3 | 21.0±0.1 | 18.8±0.5 | 11.0±0.1 | 15.3±1.0 |
| glutamic acid | 11.1±1.0 | 22.2±0.1 | 18.6±0.1 | 21.9±0.5 | 38.5±0.1 | 22.3±0.3 | 20.6±0.3 | 16.7±0.2 | 20.1±0.2 | 39.8±0.1 | 38.2±0.6 | 24.9±0.1 | 36.9±1.0 |
| proline | 22.5±0.6 | 8.4±0.1 | 9.8±0.2 | 12.7±0.3 | 16.5±0.5 | 9.5±0.2 | 11.0±0.1 | 9.3±0.9 | 8.9±0.2 | 15.0±0.4 | 15.8±0.3 | 8.5±0.2 | 15.5±0.5 |
| glycine | 10.3±1.0 | 8.2±0.2 | 7.5±0.2 | 8.1±1.1 | 9.8±0.1 | 7.5±0.6 | 9.5±0.5 | 7.5±0.1 | 7.4±0.1 | 15.7±0.3 | 16.2±0.1 | 8.7±0.1 | 14.8±0.1 |
| alanine | 9.0±0.2 | 16.4±0.3 | 7.7±0.3 | 8.5±0.4 | 12.4±0.2 | 16.3±0.3 | 15.5±0.3 | 11.8±0.1 | 11.9±0.1 | 24.8±0.3 | 24.7±0.5 | 14.1±0.1 | 22.5±0.1 |
| cysteine | 14.9±0.1 | 0.8±0.1 | 0.3±0.1 | 0.2±0.1 | 0.6±0.2 | 0.7±0.1 | 0.7±0.1 | 0.5±0.1 | 0.5±0.1 | 1.4±0.1 | 1.5±0.1 | 0.7±0.1 | 0.9±0.1 |
| valine | 0.7±0.1 | 13.5±0.1 | 9.3±0.2 | 10.5±0.6 | 14.2±0.2 | 11.3±0.4 | 13.3±0.1 | 9.5±0.1 | 10.3±0.2 | 16.6±0.4 | 21.0±0.2 | 10.5±0.2 | 18.5±0.1 |
| methionine | 12.1±0.2 | 3.4±0.1 | 1.0±0.1 | 2.1±0.6 | 2.4±0.5 | 3.3±0.1 | 2.7±0.1 | 2.1±0.3 | 2.5±0.1 | 4.4±0.1 | 5.0±0.1 | 2.7±0.1 | 4.3±0.4 |
| isoleucine | 2.7±0.1 | 14.6±0.4 | 9.3±0.3 | 9.2±0.2 | 9.5±0.3 | 22.4±0.2 | 11.7±0.2 | 0.29.5± | 14.1±0.5 | 16.3±0.4 | 23.5±0.4 | 24.3±0.2 | 19.1±0.1 |
| leucine | 15.4±0.2 | 15.7±0.1 | 8.5±0.3 | 10.2±0.1 | 14.6±0.3 | 20.2±0.1 | 16.6±0.4 | 11.3±0.1 | 15.8±0.1 | 21.3±0.1 | 23.6±0.5 | 16.1±0.1 | 21.2±0.1 |
| tyrosine | 16.5±0.3 | 6.8±0.1 | 4.2±0.2 | 3.6±0.1 | 4.5±0.4 | 6.9±0.2 | 6.6±0.2 | 4.8±0.1 | 4.7±0.1 | 9.5±0.1 | 9.8±0.2 | 5.2±0.1 | 9.9±0.1 |
| phenyloalanine | 6.4±0.1 | 9.5±0.5 | 4.6±0.5 | 5.6±0.3 | 7.2±0.6 | 8.4±0.3 | 10.5±0.2 | 7.3±0.1 | 7.2±0.1 | 12.5±0.4 | 13.8±0.2 | 7.6±0.1 | 13.5±0.1 |
| histidine | 9.2±0.1 | 4.9±0.2 | 3.4±0.1 | 4.2±1.1 | 4.2±0.1 | 5.2±0.1 | 5.3±0.1 | 3.9±0.1 | 3.9±0.1 | 7.1±0.1 | 8.4±0.1 | 4.9±0.1 | 7.4±0.1 |
| lysine | 5.1±0.1 | 13.4±0.4 | 8.3±0.3 | 10.5±0.7 | 12.4±0.3 | 14.5±0.4 | 14.3±0.4 | 10.5±0.1 | 11.8±0.4 | 20.8±0.3 | 24.4±0.3 | 15.0±0.1 | 22.8±0.1 |
| arginine | 15.1±0.2 | 8.6±0.3 | 5.8±0.2 | 6.2±0.1 | 7.6±0.2 | 8.9±0.1 | 9.8±0.2 | 7.3±.0.1 | 7.8±0.1 | 14.6±0.1 | 14.5±0.3 | 9.7±0.1 | 14.8±0.1 |
