# Supplementary material for: High value-added products derived from crude glycerol via microbial fermentation using Yarrowia clade yeast
Source: Microb Cell Fact. 2021 Oct 9;20:195. doi: 10.1186/s12934-021-01686-0 (PMC8502345; doi:10.1186/s12934-021-01686-0)
Supplement: Supplementary file 3 — Additional file 3: Table S3. Comparison of enzymatic activities of key enzymes from erythritol biosynthesis pathway from glycerol by selected stains from the Yarrowia clade. [file 12934_2021_1686_MOESM3_ESM.docx]

**Table S3.** Comparison of enzymatic activities of key enzymes from erythritol biosynthesis pathway from glycerol by selected stains from the *Yarrowia* clade

| **Strain** | **Culture phase** | **GK** | | **GPDH** | | **TK** | | **ER** | |
| --- | --- | --- | --- | --- | --- | --- | --- | --- | --- |
|  |  | **(U mg^-1^)** | **(U CDW^-1^)** | **(U mg^-1^)** | **(U CDW^-1^)** | **(U mg^-1^)** | **(U CDW^-1^)** | **(U mg^-1^)** | **(U CDW^-1^)** |
| **YAOS** | GP | 0.401 | 65.10 | 0.019 | 2.98 | 0.025 | 4.27 | 0.276 | 52.31 |
|  | SP | 0.303 | 59.76 | 0.017 | 3.37 | 0.009 | 2.13 | 0.035 | 5.39 |
| **YADI** | GP | 0.384 | 110.50 | 0.015 | 4.49 | 0.024 | 7.37 | 0.323 | 86.36 |
|  | SP | 0.373 | 105.73 | 0.019 | 4.11 | 0.023 | 5.39 | 0.267 | 73.08 |
| **YALI^a^** | GP | 0.026 | 5.35 | 0.02 | 4.18 | 0.07 | 14.47 | 0.230 | 47.79 |
|  | SP | 0.010 | 1.43 | 0.018 | 2.48 | 0.024 | 3.25 | 0.185 | 25.25 |
| GP - growth phase (24 h of culture), SP - stationary phase (at the end of culture), GK – glycerol kinase, GPDH - glycerol-3-phosphate dehydrogenase, TK – transketolase, ER - erythrose reductase, CDW – 100 mg of dry cell weight, SD (standard deviation) does not exceed 0.1, YAOS -  *Yarrowia divulgata* CBS11013, YADI -  *Yarrowia divulgata* CBS11013; ^a^Tomaszewska et al., 2014 | | | | | | | | | |
